# Prevalence of *Strongylus vulgaris* in horses after ten years of prescription usage of anthelmintics in Sweden

**DOI:** 10.1016/j.vpoa.2019.100013

**Published:** 2019-05-26

**Authors:** Eva Tydén, Heidi Larsen Enemark, Mikael Andersson Franko, Johan Höglund, Eva Osterman-Lind

**Affiliations:** a Swedish University of Agricultural Sciences, Department of Biomedical Sciences and Veterinary Public Health, Section for Parasitology, Uppsala, Sweden; b Norwegian Veterinary Institute, Department of Animal Health and Food Safety, Oslo, Norway; c Karolinska Institutet, Department of Medical Epidemiology and Biostatistics, Stockholm, Sweden; d National Veterinary Institute, Department of Microbiology, Section for Parasitology diagnostics, Uppsala, Sweden

**Keywords:** *Strongylus vulgaris*, Horse, Selective treatment, Deworming routines

## Abstract

*Strongylus vulgaris* is considered the most pathogenic nematode parasite of the horse. Frequent deworming programs since the 1970s have reduced the prevalence of *S. vulgaris* to low levels, but to the price of widespread benzimidazole resistance in the small strongyles (cyathostominae) and ivermectin resistance especially in the equine roundworm *Parascaris* spp. To slow down the progression of anthelmintic resistance in Sweden, horse anthelmintics were made prescription only medicine in 2007 and selective therapy principles were introduced. This means that only individuals with high egg excretion or clinical signs of helminth infection were treated instead of blanket treatment of all horses on a farm. The aims of this study were to investigate the prevalence and risk factors associated with *S. vulgaris* infection in Sweden ten years after the introduction of a selective therapy regime. A total of 529 faecal samples from 106 farms were collected during March to June in 2016 and 2017. A web-based questionnaire was used to collect information about deworming routines. Strongyle faecal egg counts (FEC) were performed by McMaster and presence of *S. vulgaris* was demonstrated with a specific PCR on individual larval cultures. Results were analysed by mixed logistic (*S. vulgaris* prevalence) or linear (FEC) regression models, where farm was included as random factor. The overall prevalence of *S. vulgaris* at individual and farm levels was 28% and 61%, respectively. We observed a 2.9 increased odds risk of *S. vulgaris* infection on farms that based their treatment on strongyle FEC only as compared to farms that complemented strongyle FECs with larval cultures or dewormed regularly, 1–4 times per year, without prior diagnosis. We found no association between the prevalence of *S. vulgaris* and strongyle FEC level, horse age, geographical region or signs of colic. The prevalence of *S. vulgaris* was 25% in horses shedding ≤150 eggs per gram. Thus horses with low strongyle FECs that are left untreated could be an important source of *S. vulgaris* infection. This may be an important reason for the approximately three-fold increase in *S. vulgaris* prevalence since 1999 in Sweden. However, our combined results indicate that selective therapy based on a combination of strongyle FECs and larval cultivation was not associated with an increased risk of *S. vulgaris* infection. Still, *S. vulgaris* needs to be monitored continuously and should be taken into careful consideration when the treatment frequency is reduced.

## Highlights

• Three-fold increase of *Stronylus vulgaris* prevalence in horses in Sweden.
• 2.9 increased odds risk of *S. vulgaris* on farms only performing faecal egg counts.
• No association between the prevalence of *S. vulgaris* and egg counts or horse age.
• No association between the prevalence of *S. vulgaris* and signs of colic on the farms.

## Graphical abstract

**Figure:**
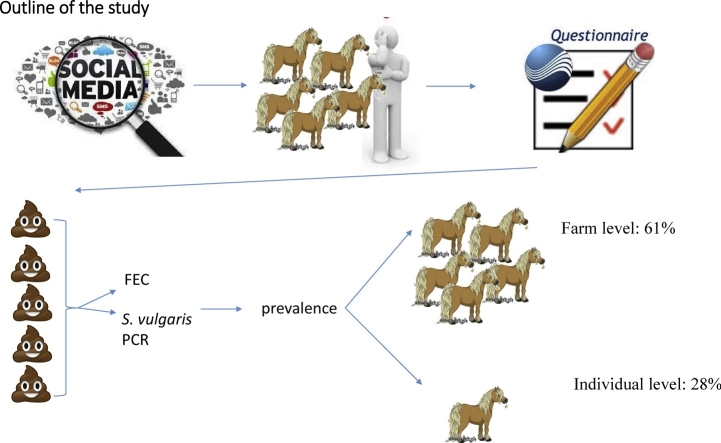

## 1 Introduction

The equine industry has expanded in Sweden and the number of horses is estimated to 350 000, which is comparable with the number of dairy cows. Approximately 80% of the horse farms are located close to cities (Enhäll, 2017) where extensive grazing areas are limited allowing accumulation of parasite eggs in the paddocks.

Strongyle nematodes are ubiquitous parasites of the horse with the large strongyle *Strongylus vulgaris* being considered the most pathogenic species (Duncan, 1974). The life cycle of *S. vulgaris* is completed within 6–7 months and involves migration of larvae to the cranial mesenteric arteries where they grow in size and develop during 3–4 months before they migrate downstream to enter the lumen of the cecum/colon (Duncan, 1974). The migrating larvae induce endarteritis in the mesenteric arteries, which may provoke thickening of the arterial wall, thrombus formation, infarction and necrosis. The term “thrombo-embolic colic” refers to the severe clinical signs caused by detachment of thrombus material, which is carried downstream to obliterate smaller arteries or arterioles causing ischemia and infarction of the corresponding intestinal segment (Duncan and Pirie, 1975).

In the 1960-80 s, *S. vulgaris* was widespread with a prevalence of 80–100% on farm level in the USA and Scandinavia (Slocombe and McCraw, 1973, Nilsson and Andersson, 1979, Tolliver et al., 1987). When broad-spectrum anthelmintic drugs were introduced in the 1970-80 s, prophylactic strategies were usually employed including several treatments per year aiming at interrupting the life-cycle of *S. vulgaris* before the female worms started to produce eggs (Love and Duncan, 1991, Drudge and Lyons, 1966). This approach was successful in controlling *S. vulgaris* infection and the prevalence in Scandinavia decreased to around 5% at the individual level (Craven et al., 1998; Lind Osterman et al., 1999). Unfortunately, these frequent treatments resulted in the development of resistance against benzimidazole in small strongyles and against ivermectin in the large roundworms *Parascaris* spp. and problems with multidrug resistance have been observed in both Europe and USA in both parasite species (reviewed by Peregrine et al., 2014, Martin et al., 2018).

To reduce the selection pressure for drug resistance in the parasites selective therapy was introduced in the early 2000′s. This approach is based on anthelmintic treatment of individual horses with a strongyle faecal egg count (FEC) exceeding a chosen cut-off value, often 200 eggs per gram faeces (EPG) (Uhlinger, 1993). This strategy was first developed for ruminants where the problems with anthelmintic resistance are more critical (reviewed by Kenyon et al., 2009). In horses, selective therapy was first implemented in 1999 in Denmark and then shortly followed by Sweden and the Netherlands (Nielsen, 2012).

The Swedish National Veterinary Institute (Swedish National Veterinary Institute, 2018) recommends faecal analyses of all horses before turnout in the spring and after the grazing season in the autumn. Deworming is suggested only for horses with a strongyle EPG above 200 and/or positive for *S. vulgaris* and/or the equine tapeworm *Anoplocephala perfoliata*. Unfortunately, there is a risk that not all horse owners ask for extended analyses of *S. vulgaris* and *A. perfoliata* due to economic factors or ignorance, which could lead to neglect of *S. vulgaris* infections. The implementation of prescription only of anthelmintic drugs has contributed to a drastic reduction anthelmintics sold to horses and livestock in Sweden. In 2007, the year before prescription anthelmintic drugs to horses and livestock was implemented by the Swedish Medical Products Agency, 3645 kg macrocyclic lactones, benzimidazoles and pyrantel were sold in Sweden, whereas in 2016, only 1445 kg of these drugs were sold (Girma, 2016). However, deworming without faecal analysis is allowed in Sweden if the veterinarian is well informed about the management on the farm (SJVFS, 2010:17 24 §).

In Sweden, the most recent nationwide study describing the prevalence of *S. vulgaris* was conducted in 1999, 8 years before the selected treatment regime was advocated to horse owners. That study showed a herd prevalence of 14% based on morphological identification of larvae on 110 farms recruited from a list of riding schools at the Swedish Equestrian Federation and stud farms at the Breeding Association in Sweden (Lind Osterman et al., 1999). Today many farms/horse owners in Sweden regularly submit samples for coproscopic parasitological analysis. However, not all of them ask specifically for *S. vulgaris* or *A. perfoliata* identification, which possibly leads to underreporting of these parasites.

The objectives of our study were to: (i) investigate the prevalence of *S. vulgaris* after the implementation of selective therapy in Sweden; (ii) analyse possible risk factors for *S. vulgaris*; (iii) obtain information about deworming routines applied on Swedish horse farms.

## 2 Materials and methods

### 2.1 Farms

The study was performed during two consecutive years from March to June in 2016 and 2017. The study was announced in social media such as the Facebook pages of the Swedish National Veterinary Institute (SVA), the Swedish University of Agricultural Sciences (SLU) and “HästSverige” (a science-based website directed to horse owners). In this way 20 horse farms were recruited each year from the three regions in Sweden: south, central and north. The inclusion criteria were: (i) a farm size of at least five horses; (ii) animal age minimum 2 years; (ii) no anthelmintic treatment performed within six months prior to sampling; and (iii) response to a web-based questionnaire about deworming routines (Table 1). The first 20 farms from each region that fulfilled the inclusion criteria were included in the study.

**Table 1:** Questionnaire data collected on the participating horse farms.

Information	Descriptor
Farm location	Zip code
Age of horses	Years
Time since last anthelmintic treatment	Months < 3; < 6; < 12; < 24; < 36; ≤ 48
Anthelmintic	Drug used at last treatment
Signs of colic^a^ last 24 months	Yes/No
Tested positive for *Strongylus vulgaris* during the last 24 months	Yes/No
Deworming routines applied on the farm	i) only after FEC^b^ ii) after FEC and cultivation for *S. vulgaris* iii) routine deworming 1-4 times/ year

^a^ restlessness and pawing at the ground, irritated kicking to the stomach, rolling or attempting to roll ^b^ faecal egg count.

### 2.2 Faecal samples

After completing the questionnaire, faecal samples from five randomly selected horses older than two years on each farm were collected by the owners or staff working in the stable and packed according to the recommendations from the Swedish National Veterinary Institute (2018). Samples were then sent by regular mail to the parasitology laboratory of SLU. Upon arrival, the samples were stored at 4 °C until analysis within two days. Strongyle FECs were carried out for each horse using a modified McMaster technique with a theoretical sensitivity of 50 EPG (Coles et al., 1992). Nematode eggs in faecal samples (3 g) were flotated using a saturated NaCl solution (SG = 1.18) (Coles et al., 1992).

### 2.3 Strongylus vulgaris detection

Irrespective of FEC, larval cultures for detection of *S. vulgaris* were performed on 50 g faeces from each horse according to Bellaw and Nielsen (2015). In brief, faeces were mixed with an equal volume of vermiculite (Weibulls, Sweden), tap water was added to obtain a moist condition and samples were cultured at 20 °C for 14 d. Third stage larvae were harvested after sedimentation for 12–16 h at 20 °C by the inverted Petri dish method (Van Wyk and Mayhew, 2013).Pellet of harvested L3 was performed by collecting approximately 20 ml of the fluid into a 50 ml Falcon tube, centrifuged at 248 x *g* for 3 min and discarded the supernantant. Larval DNA was extracted prior exsheathment with NuceloSpin^®^Tissue (Macherey-Nagel, Germany) according to the manufacturer´s instruction. Theconcentration and quality of the DNA were analysed using Pico200 (Picodrop, United Kingdom). Samples were then screened for *S. vulgaris* by real time PCR using the Rotorgene 3000 system with QuantitecTect^®^SYBR^®^ Green PCR kit (Qiagen, Germany). Primers were synthesized by Eurofins Genomics (Germany) according to Nielsen et al. (2008) to amplify a 171 PCR product of the second internal transcribed spacer (ITS-2) of *S. vulgaris* (accession number in GenBank: X77863.1); forward primer 5`GTATACATTAAATTGTGTCCCCCATTCTAG3` and reverse primer 5`TTGCAAATATCATTAGATTTGATTCTTCCG3`. The 25 μl reaction volume consisted of 5 μl template DNA, 0.5 μM of each primer and 12.5 μl 2xQuantiTec SYBR Green PCR master mix. The following PCR protocol was used: 95 °C for 15 min followed by a set of 40 cycles, 94 °C for 15 SI, 55 °C for 30 SI and 72 °C for 30 SI ended with melt curve analysis to verify the specificity and identity of the PCR products. A non-template control and DNA extracted from an adult *S. vulgaris* were run for each reaction. Samples were considered as positive with a threshold value (Ct) <38 with a specific melt curve analyses. Samples with a Ct-value ≥38 were verified to be positive after Sanger sequencing using Macrogen Europe (Netherlands). PCR products were prepared for sequencing usingExoSAP-IT™ PCR Product Cleanup Reagent (ThermoFisher) to excess primers and nucleotides.

### 2.4 Statistical analyses

Before statistical analysis, strongyle FECs were clustered into the following EPG groups: 0; 50–150; 200–500; 550–800; ≥850 and animal age into the following categories: 0–5; 6–10; 11–15; 16–20 and ≥ 21 years of age. Individual prevalence of *S. vulgaris* in relation to year of the study, geographic region, age, EPG, time since last deworming, deworming routine applied on the farm, colic within 24 months prior to sampling and positive for *S. vulgaris* within 24 months was analysed using a mixed logistic regression model with farm as random factor. Confidence intervals (95%) were calculated using bootstrap sampling simulation of farms to account for the clustering effect. Strongyle FEC in relation to year of the study, region, age, EPG, deworming routine applied on the farm, time since last deworming and drug used at last treatment was analysed using a mixed linear regression model with farm as random factor. EPG was logarithmic transformed to handle the skewed distribution. Individual horse treatments were not included in the mixed logistic regression model. The model fit was assessed by residual plots to determine normally distributed residuals. Since the detection limit was 50 EPG, all values below this threshold were replaced by the midpoint 25. The software R version 3.4.1 was used for all statistical calculations using the lmer function in the lme4 package in the mixed logistic regression and the lme function in the nlme package in the mixed linear regression (R Core Team (2017).). All statistical analyses were interpreted as statistically significant up to p-value ≤ 0.05 and a confidence interval (CI) of 95%.

## 3 Results

### 3.1 Farms

In total, 529 horses from 106 farms participated in the study; 39 farms from the south, 40 farms from the central and 27 farms from the north of Sweden. The mean age of the horses was 11.6 (SD ± 6.3) years and time since latest deworming ranged from 6 to 48 months, with a mean of 16 (SD ± 12.8) months and a median of 12 months.

### 3.2 Prevalence of Strongylus vulgaris

Examination of 529 individual larval cultures by PCR showed an overall *S. vulgaris* prevalence of 28% (95% CI: 22–33%) with a mean of 1.4 positive horses out of five examined per farm. No significant differences were found between the years (p = 0.15); 32% (95% CI: 24–39%) in 2016, and 24% (95% CI: 16–31%) in 2017. The overall prevalence of *S. vulgaris* at the farm level was 61% (95% CI: 51–70%, 65 of 106 farms), and no significant differences (Table 2) were found between the three regions; 64% (95% CI: 47–79%) in south, 60% (95% CI: 43–75%) in central, and 59% (95% CI: 39–78%) in northern Sweden.

**Table 2:** Logistic regression of individual *Strongylus vulgaris* prevalence in Swedish horses against individual risk factors for infection.

Investigated parameter		Odds ratio (95% ^a^C.I.)	p-value
Year of the study	2016	REF ^b^	0.15
	2017	0.6 (0.3-1.2)	
Region	South	REF	0.67
	Central	0.9 (0.4-2.1)	
	North	0.6 (0.2-1.7)	
Age group	0-5	REF	0.84
	6-10	0.7 (0.3-1.6)	
	11-15	0.8 (0.3-1.6)	
	16-20	0.8 (0.4-1.9)	
	>21	0.6 (0.2-1.5)	
EPG^c^	<50 50-150	REF 0.7 (0.3-1.4)	0.44
	200-450	0.7 (0.3-1.5)	
	500-800	1.3 (0.6-2.9)	
	≥ 850	1.1 (0.5-2.6)	
Time elapsed since deworming	6 months	REF	0.51
	8 months	0.6 (0.2-2.1)	
	10 months	0.6 (0.2-2.0)	
	12 months	0.7 (0.3-1.8)	
	24 months	1.7 (0.5-5.5)	
	48 months	1.1 (0.3-3.8)	
Deworming routine	FEC^d^	REF	0.044
	FEC and cultivation for *S. vulgaris*	0.3 (0.1-0.8)	
	Deworming 1-4 times/ year	0.5 (0.2-1.3)	
Colic last 24 months ^e^	yes	REF	0.94
	no	1.0 (0.4-2.2)	
*S. vulgaris* positive last 24 months	yes	REF	0.13
	no	1.9 (0.7-5.8)	
	not cultivated	2.9 (1.0-8.9)	

^a^Confidence interval ^b^ reference sample used to calculate odds ratio within each investigated parameter ^c^ nematode eggs per gram ^d^ faecal egg count ^e^ if the horse had shown any signs of restlessness and pawing at the ground, irritated kicking to the stomach, rolling or attempting to roll during the last 24 months.

**Fig. 1:**
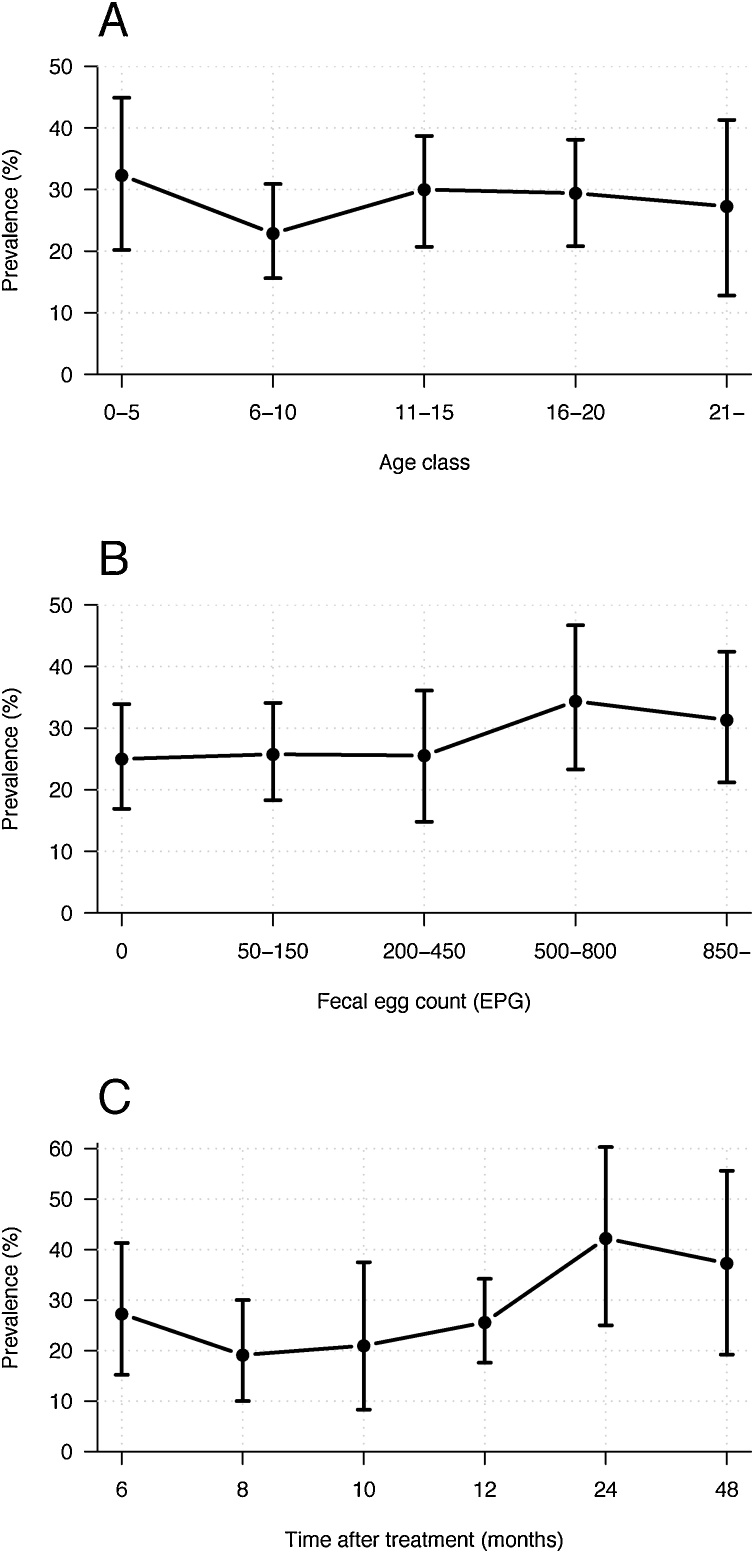
Prevalence of Strongylus vulgaris A) in relation to the age of the horses. The horses were clustered in age groups 0–5; 6–10; 11–15; 16–20 and ≥ 21 years., B) in relation to faecal egg counts clustered as follow: 0; 50–150; 200–450; 500–800; ≥850 eggs per g of faeces (EPG). C) in relation to last deworming. The vertical lines in A–C indicate the 95% confidence intervals.

Patent *S. vulgaris* infection was found in all age groups and was not significantly correlated to strongyle FEC levels (Fig. 1A, B). Prevalence of *S. vulgaris* in this study population in relation to latest deworming was based on questionnaire data provided by the horse owners. The prevalence tended to be positively correlated with time since the last deworming (Fig. 1 C), but this was not statistically significant (Table 2). A summary of the results is presented in Table 2. The only significant risk factor found was the deworming decision applied on the farms. Hence, no association was found between signs of colic within a period of 24 months prior to sampling or a positive *S. vulgari*s test outcome.

### 3.3 Strongyle faecal egg counts

**Fig. 2:**
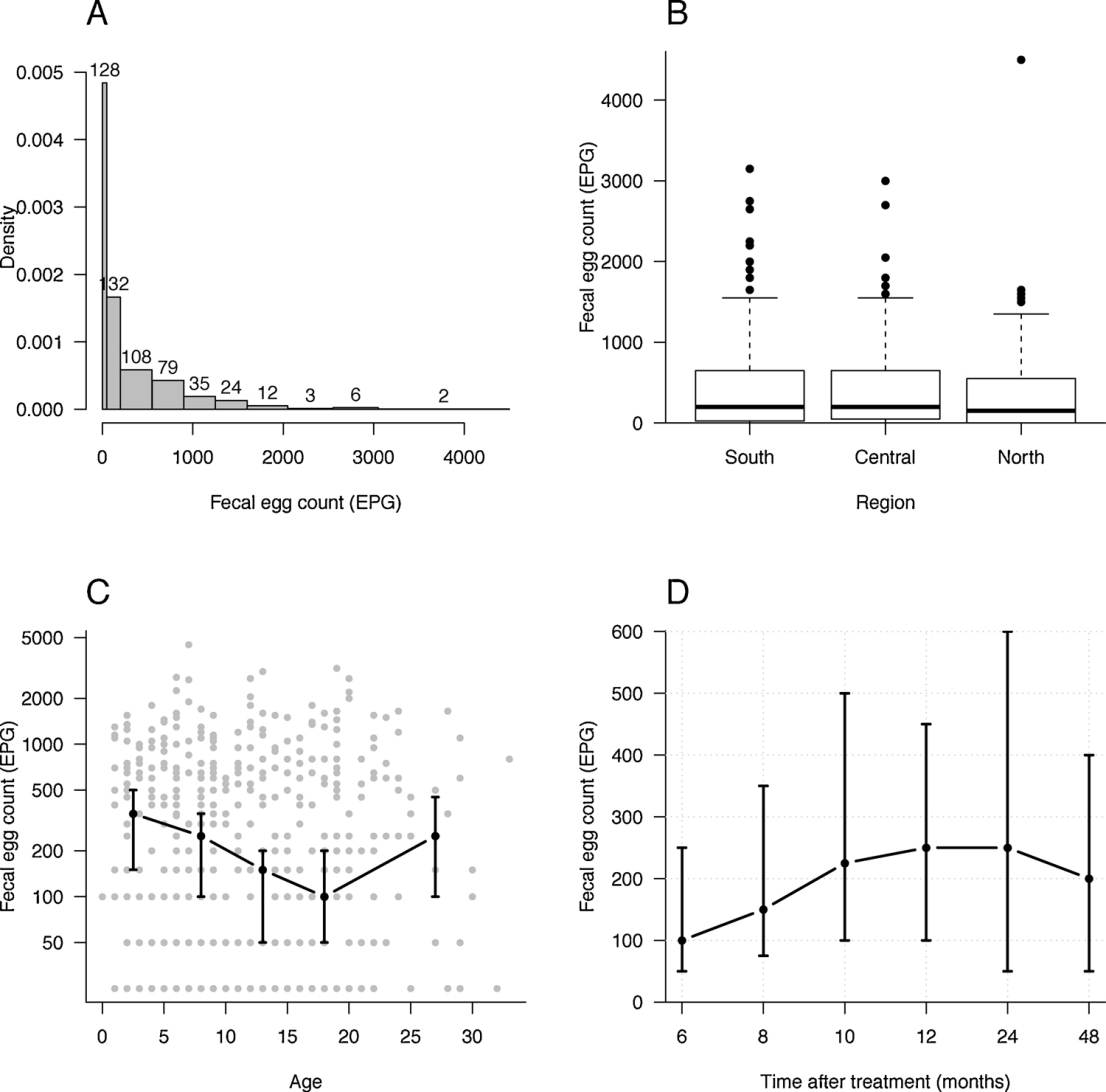
A) A density histogram reflecting the skewed distribution of eggs per g of faeces (EPG) where the width of the bar represent the size of each EPG-cluster. EPG were clustered as follow: 0; 50–150; 200–500; 550–850; 900–1200; 1250–1550; 1600–2000; 2050–2500; 2550–3000; 3050-4500. Figures above each bar indicate number of horse in each EPG-cluster. Density of the y-axis illustrates the number of horses divided by the length of each corresponding interval. For example, there were 529 horses in total of which 128, corresponding to 24%, had EPG < 50. The density of this class is hence 0.24/50 = 0.0048. B) A boxplot of EPG in the three different regions in Sweden. The bold line indicates median, the fine lines are the quartiles and dots represents outliers in the population and the horizontal line in the boxplot is the so called ‘upper whisker’. There was no statistical differences in EPG levels between the regions. C) Median EPG levels (black dots) plotted against the age of the horses. The horses are clustered in age groups of 0–5; 6–10; 11–15; 16–20 and ≥ 21 years. The dots represent the median of each age group and the vertical lines indicate the 95% confidence intervals. All data are included as grey dots. The Y-axis is log transformed. D) Median EPG in relation to last treatment. The vertical lines indicate the 95% confidence intervals.

The strongyle FECs varied from <50 to 4500 EPG. At least one horse on each of the 106 farms included in the study had a FEC of ≥50 EPG. Of the 529 horses 401 (76%) were shedding strongyle eggs. The main proportion of the horses in the study were shedding less than 250 EPG, with 24% excreting below 50 EPG and 25% between 50 and 200 EPG. Fifty-one per cent of the horses were shedding >200 EPG and of these 8% had ≥1600 EPG (Fig. 2 A).

The mean strongyle FECs in the different regions were 463(SD ± 610) EPG in the south, 420(SD ± 530) EPG in the central and 362(SD ± 550) EPG in the north of Sweden, but they were not significantly different between the three regions (Fig. 2B). In contrast, a significant (p = 0.016) difference in strongyle FEC was found between age groups (Table 3). The number of strongyle eggs was highest in horses younger than 5 years where the median EPG was 350 (95% CI: 150-500). It then declined with increasing age and horses 16-20 years of age had a median of 100 EPG (95% CI: 50-200). The strongyle FEC increased to a median EPG of 250 (95% CI: 100-450) in horses older than 20 years (Fig. 2C). There was no correlation between EPG levels and the time from the last anthelmintic treatment (Fig. 2 D). The statistical analyses, using a mixed linear regression model with a logarithmic transformation of EPG to test strongyle FECs versus various parameters, are summarized in Table 3.

**Table 3:** Linear regression of faecal egg count against individual risk factors for *Strongylus vulgaris* infection.

Investigated parameter		Rel. effect (95% ^a^C.I.)	p-value
Year of the study	2016	REF *	0.79
	2017	1.0 (0.7-1.5)	
Region	South	REF	0.49
	Central	1.0 (0.7-1.5)	
	North	0.8 (0.5-1.2)	
Age group	0-5	REF	0.016
	6-10	0.8 (0.5-1.1)	
	11-15	0.6 (0.4-0.8)	
	16-20	0.5 (0.4-0.8)	
	21-	0.7 (0.4-1.1)	
Time since deworming	6 months	REF	0.84
	8 months	1.2 (0.7-2.1)	
	10 months	1.3 (0.7-2.3)	
	12 months	1.3 (0.8-2.1)	
	24 months	1.2 (0.6-2.2)	
	48 months	1.0 (0.6-1.8)	
Anthelmintic drug	IVM^b^	REF	0.66
	BZ^c^	2.0 (0.5-7.9)	
	MOX^d^	1.3 (0.9-2.1)	
	PYR^e^	1.0 (0.4-2.4)	
Deworming routine	FEC only	REF	0.23
	FEC and cultivation for *S. vulgaris*	0.8 (0.6-1.2)	
	Deworming 1-4 times/year	1.2 (0.8-1.9)	

^a^confidence interval ^b^ reference sample used to calculate odds ratio within each investigated parameter ^b^ ivermectin ^c^ benzimidazole ^d^ moxidectin ^e^ pyrantel.

### 3.4 Deworming routines

Routine deworming 1–4 times per year without coproscopic analysis was done in 22 out of 106 farms (21%)(Table 1)., Selective therapy based on information from strongyle FECs was performed on 51 out of 106 farms (48%), and selective therapy based on both strongyle FECs and larval cultivation was used on 33 out of 106 farms (31%). The mean number of months that elapsed following the last treatment was 11.6 on farms that dewormed on a regular basis, 12.4 on farms that performed strongyle FECs only, and 15.5 on farms that performed strongyle FECs and cultivation for strongyle larvae.

A significant association (p = 0.044) was found between the prevalence of *S. vulgaris* and deworming routines with 2.9 times (95% CI: 1.2–6.9) higher odds of infection on farms using selective therapy based on strongyle FECs only compared to farms that dewormed routinely and farms combining strongyle FECs and larval cultivation. *S. vulgaris* prevalence was highest on farms only performing strongyle FECs; 35% (95% CI: 26–43%) and 73% (95% CI: 59%–85%) at individual and herd levels, respectively. The lowest prevalence was observed on farms that applied both strongyle FECs and larval cultivation; 21% (95% CI: 12–31%) at the individual level and 44% (95% CI: 27–62%) at the herd level. The corresponding figures on farms that dewormed routinely without a diagnosis was 24% (95% CI: 14–35%) at the individual level and 61% (95% CI: 39–80%) at the herd level. The median EPG was 200 on farms that based deworming on strongyle FECs; 150 on farms that used both strongyle FECs and larval cultivation, and 350 on farms that dewormed routinely, but these figures were not significantly different (p = 0.44).

Macrocyclic lactones was the anthelmintic group mostly used (73%, n = 77). Of these farms, 50% used ivermectin, 14% moxidectin, and 9% used a combination of ivermectin/moxidectin and praziquantel. Only 3% used pyrantel and 1% used fenbendazole. As many as 23% of the farmers did not remember which drug they had used.

## 4 Discussion

This study demonstrates anincrease of *S. vulgaris* prevalence (28% at individual and 61% herd levels) in Sweden since the last survey in 1999 (14% at herd level) (Lind Osterman et al., 1999). The higher prevalence reported here compared to 1999 (Lind Osterman et al., 1999) might to a certain extent be explained by a different inclusion criterion regarding the time since last deworming, but also methodological differences. Considering the long lifecycle of *S. vulgaris*, only horses whiteout treatment during the last six months were included in the present study, whereas the corresponding time was two months in Lind et al. (1999). More importantly though, in this study were all samples analysed individually compared to pooled samples from four horses in 1999 (Lind Osterman et al., 1999). In addition, was *S. vulgaris* diagnosed with a specific PCR, which is considered to be a more sensitive method than the traditional morphological identification of harvested larvae from cultures (Kaspar et al., 2017). However, a relatively recent study from Denmark also found a high prevalence of *S. vulgaris* although the results were based on morphological identification of larvae (Nielsen et al., 2012). The overall prevalence of *S. vulgaris* in this neighboring country was 12% and 64% on individual and herd levels, respectively, which agree with our results. Moreover, results from the routine diagnostic analyses at the Swedish National Veterinary Institute also show an increase in the number of *S. vulgaris* positive horses from 6% in 2008 to 32–53% in the years 2013–2017 based on morphological analyses (Swedish National Veterinary Institute, unpublished results).

Since 2008 anthelmintic drugs have been used in Sweden exclusively by prescription of a licensed veterinarian according to an EU directive (EUR-Lex, 2018), which has led to more than 50% reduction of anthelmintics sold to horses and livestock in the subsequent 10 year period (Girma, 2016). Thus, less anthelmintics are used today not only on farms that perform selective therapy, but also on farms that deworm without prior diagnosis. In the present study, farms deworming on a regular basis treat on average once a year as compared to 3.1 treatments per year according to questionnaire data from 2007 (Lind Osterman et al., 2007). Standardized treatment programs applied to horses soon after the introduction of modern broad spectrum anthelmintics sometimes involved 4–6 treatments per year (Drudge and Lyons, 1966). Considering that the prepatent period of *S. vulgaris* is 6–7 months, farms that do not perform diagnostics would need to deworm twice yearly (i.e late spring and autumn/winter) to keep the pasture free of *S. vulgaris* larvae. In our study, *S. vulgaris* was still detected on farms that dewormed routinely on a yearly basis without performing any parasite diagnostics.

It is generally recommended to horse owners that control strategies should be based on analysis of faecal samples (Swedish National Veterinary Institute, 2018). However, correct implementation of selective therapy should include not only regular monitoring with strongyle FEC tests but also specific analysis of *S. vulgaris* (Nielsen et al., 2012, Swedish National Veterinary Institute, 2018). We believe that many horse owners use selective therapy based solely on strongyle FECs while they are not fully aware of the need of requesting additional specific analysis for *S. vulgaris*. According to our study, horses with no or few nematode eggs are often left untreated and these animals may then potentially contaminate the grazing areas with *S. vulgaris* eggs. This could likely be one reason for the high prevalence of *S. vulgaris* observed in this study. Our finding that farms, which based their anthelmintic treatments on strongyle FECs were 2.9 times more likely to be infected with *S. vulgaris* compared to those that dewormed routinely or included testing for *S. vulgaris* in the control program further supports this hypothesis. Similarly, in Denmark a higher prevalence of *S. vulgaris* was observed on farms using selective treatment compared to farms deworming on a regular basis (Nielsen et al., 2012). In contrast, low prevalences of *S. vulgaris* (0.2–1.3%) have been reported from Germany (Schneider et al., 2014, Kaspar et al., 2017). However, selective treatment is not implemented as strict in Germany compared to Sweden and Denmark and many horses are still routinely dewormed (Schneider et al., 2014).

It should be noted though that farms included in our study were not randomly selected, which could have influenced the results. The Swedish Board of Agriculture has a record of registered horses in Sweden, but not of all farms, therefore, social media were used to recruit farms. This strategy could have biased the results as the participating farms may perhaps have had a particular interest in parasites due to high infection pressure or recurring *S. vulgaris* infection on the farms. Alternatively, farms with a particular interest in parasites or the wellbeing of their horses may have submitted more samples for parasitological diagnostic analysis and therefore have had fewer parasites than average Swedish horse farms. Nevertheless, although a truly random sampling could not be done due to the absence of a register of all horse farms, we consider our sampling representative for the whole country. Even; if there is a risk of a somewhat biased sampling our results demonstrate a dramatic increase in the *S. vulgaris* prevalence compared to the Swedish study conducted twenty years ago (Lind Osterman et al., 1999).

In accordance with Nielsen et al. (2012) the prevalence of *S. vulgaris* was not associated with faecal nematode egg output or horse age. Twenty-five per cent of the horses with egg excretion below the detection level or low egg excretion (0-150 EPG) were positive for *S. vulgaris;* thus using a cut-off level of 200 EPG as a criterion for deworming would mean that such horses will not be dewormed. Thus, a key conclusion of our study is that not only horses with high egg excretion, but also horses with low egg excretion should be examined for *S. vulgaris.*

In 2007, only 1% of horse farms in Sweden submitted faecal samples on a regular basis for parasitological analysis at a diagnostic laboratory (Lind Osterman et al., 2007). In the present study 79% of the farms used selective therapy based on diagnostic analyses, but most of them (48%) requested strongyle FEC only. The use of selective therapy varies in different countries. In Germany 44% of 195 farms dewormed based on strongyle FEC analyses (Schneider et al., 2014), whereas only 10% of 213 farms in Italy requested a coproscopic examination prior to treatment (Papini et al., 2015). In contrast, in Denmark the majority of horse farms use selective treatment due to national legislation, and according to a questionnaire by Nielsen et al. (2006), 97% of equine veterinary practitioners prescribed selective treatment. A recent national survey from the United States showed that only 22% of 380 farms from 28 states based their deworming practice on strongyle FECs (Nielsen et al., 2018). This demonstrates that deworming strategies varies substantially between countries. Selective therapy usually reduces the total number of anthelmintic treatments and thereby also the selection pressure for the development of anthelmintic resistance (Duncan and Love, 1991, Kaplan and Nielsen, 2010). Due to the risk of development of anthelmintic resistance it is not desirable to return to routine treatment strategies. To prevent further spread of *S. vulgaris*, it is therefore important to include specific tests to detect this parasite in the selective therapy program.

Of the drug classes used in Swedish horse farms the macrocyclic lactones were most commonly used (73% of the farms) according to the questionnaire data. This is in accordance with findings from the last survey in Sweden in 1999 (Lind Osterman et al., 1999) as well as two more recent studies from Denmark and Germany (Nielsen et al., 2012, Schneider et al., 2014). The spread of resistance in cyathostomine parasites is evident for benzimidzoles and pyrantel (reviewed in Peregrine et al., 2014), which has resulted in a shift to a more one sided usage of macrocyclic lactones. There is still little evidence for widespread strongyle resistance to the macrocyclic lactones in Europe, but several reports about emerging resistance with a reduction of egg reappearance period after treatment with ivermectin/moxidectin (von Samson-Himmelstjerna et al., 2007; van Doorn et al., 2014; Molena et al., 2018). However, although the macrocyclic lactones are reported to be effective against migrating *S. vulgaris* larvae (Slocombe and McCraw, 1981) we detected high numbers of *S. vulgaris* positive horses on farms that claimed to deworm on a regular basis. This may indicate reduced anthelmintic efficacy, but it could also be due to the fact that deworming was performed too sparsely; the mean interval between treatments in our study was 11.6 months compared to 9.6 months in Denmark and approximately 4–8 months in Germany (Hinney et al., 2011, Nielsen, 2012, Schneider et al., 2014). Moreover, the timing of anthelmintic treatments is an important factor in parasite control programs. Most farms in our study dewormed their horses in the spring to reduce contamination of parasite larvae on the summer pasture. Remarkably, nearly one out of four farms did not remember which drug they used at the latest deworming occasion.

Even though *S. vulgaris* is considered the most pathogenic nematode parasite in horses (Duncan, 1974) no association was found from the questionnaire between signs of colic during the 24 months and positive *S. vulgaris* analysis.

## 5 Conclusion

Many horse owners and veterinarians in Sweden have adopted a selective deworming strategy, and since anthelmintic drugs to horses became available on prescription only, in 2008, markedly less anthelmintics have been sold. In parallel, the prevalence of *S. vulgaris* has increased approximately three times, which at least partly appears to be associated with parasite analyses based on only FEC without *S. vulgaris* diagnostics. Our results show that selective therapy based on a combination of strongyle FECs and larval cultures was not associated with an increased risk of *S. vulgaris* infection compared to regular blanket treatment 1–4 times per year. Interestingly, less anthelmintic treatments were undertaken on farms performing strongyle FEC and larval cultures with a mean treatment interval of 15.5 months compared to farms that based treatment on only strongyle FECs. The only risk factor for infection with *S. vulgaris* in our study was selective therapy based on strongyle FECs alone. A key message to horse owners and veterinarians is the importance of including specific diagnostics for *S. vulgaris* even in situations when the excretion of strongyle eggs is low or below the detection limit in individual horses.

## Funding

This study was supported by the Foundation for Swedish and Norwegian Equine ResearchH-15-47-097.

## Declarations of interest

None.

## References

[bib0005] Bellaw L.J., Nielsen K.M. (2015). Evaluation of Baermann apparatus sedimentation time on recovery of *Strongylus vulgaris* and *S. edentatus* third stage larvae from equine coprocultures. Vet. Parasitol..

[bib0010] Craven J., Bjørn H., Henriksen S.A., Nansen P., Larsen M., Lendal S. (1998). Survey of anthelmintic resistance on Danish horse farms, using 5 different methods of calculating faecal egg count reduction. Equine Vet. J..

[bib0015] Coles G.C., Bauer C., Borgsteede F.H., Geerts S., Klei T.R., Taylor M.A., Waller P.J. (1992). World Association for the Advancement of Veterinary Parasitology (W.A.A.V.P.) methods for the detection of anthelmintic resistance in nematodes of veterinary importance. Vet. Parasitol..

[bib0020] Drudge J.H., Lyons E.T. (1966). Control of internal parasites of the horse. J. Am. Vet. Med. Assoc..

[bib0025] Duncan J.L. (1974). *Strongylus vulgaris* infection in the horse. Vet. Rec..

[bib0030] Duncan J.L., Pirie H.M. (1975). The pathogenesis of single experimental infections with *Strongylus vulgaris* in foals. Res. Vet. Sci..

[bib0035] Duncan J.L., Love S. (1991). Preliminary observations on an alternative strategy for the control of horse strongyles. Equine Vet. J..

[bib0040] Enhäll J. (2017). Hästar och anläggningar med häst 2016. Statistiska meddelanden. http://www.jordbruksverket.se/omjordbruksverket/statistik/statistikomr/lantbruketsdjur/arkivstatistiklantbruketsdjur.

[bib0045] EUR-lex (2018). 2001/82/EC. https://eur-lex.europa.eu/legal-content/EN/TXT.

[bib0050] Girma K. (2016). Försäljning av djurläkemedel. http://www.jordbruksverket.se/amnesomraden/djur/djurhalsopersonal/lakemedelfordjur/tillhandahallandeavlakemedel.

[bib0055] Hinney B., Wirtherle N., Kyule M., Miethe N., Zessin K., Clausen P. (2011). A questionnaire survey on helminth control on horse farms in Brandenburg, Germany and the assessment of risks caused by different kinds of management. Parasitol. Res..

[bib0060] Kaplan R.M., Nielsen M.K. (2010). An evidence-based approch to equine parasite control: it ain´t the 60s anymore. Equine Vet. Educ..

[bib0065] Kaspar K., Pfister K., Nielsen M.K., Silaghi C., Fink H., Scheuerle M.C. (2017). Detection of *Strongylus vulgaris* in equine faecal samples by real-time PCR and larval culture – method comparison and occurrence assessment. BMC Vet. Res..

[bib0070] Kenyon F., Greer A.W., Coles G.C., Cringoli G., Papadopoulos E., Cabaret J., Berrag B., Varady M., Van Wyk J.A., Thomas E., Vercruysse J., Jackson F. (2009). The role of targeted selective treatments in the development of refugia-based approaches to the control of gastrointestinal nematodes of small ruminants. Vet. Parasitol..

[bib0075] Lind Osterman E., Höglund J., Ljungström B.-L., Nilsson O., Uggla A. (1999). A field survey on the distribution of strongyle infections of horses in Sweden and factors affecting faecal egg counts. Equine Vet. J..

[bib0080] Lind Osterman E., Rautalinko E., Uggla A., Waller P.J., Morrison D.A., Höglund J. (2007). Parasite control practices on Swedish horse farms. Acta Vet. Scand..

[bib0085] Love S., Duncan J.L. (1991). Could the worms have turned?. Equine Vet. J..

[bib0090] Martin F., Höglund J., Bergström T.F., Karlsson Lindsjö O., Tydén E. (2018). Resistance to pyrantel embonate and efficacy of fenbendazole in *Parascaris univalens* on Swedish stud farms. Vet. Parasitol..

[bib0095] Molena R.A., Peachey L.E., Di Cesare A., Traversa D., Cantacessi C. (2018). Cyathostomine egg reappearance period following ivermectin treatment in a cohort of UK Thoroughbreds. Parasit. Vectors.

[bib0100] Nilsson O., Andersson T. (1979). *Stronylus vulgaris* hos häst – epizootologi och profylax. Svensk Veterinärtidning.

[bib0105] Nielsen M.K., Monrad J., Olsen S.N. (2006). Prescription-only anthelmintics - a questionnaire survey on strategies for surveillance and control of equine strongyles in Denmark. Vet. Parasitol..

[bib0110] Nielsen M.K., Peterson D.S., Monrad J., Thamsborg S.M., Olsen S.N., Kaplan R.M. (2008). Detection and semi-quantification of *Strongylus vulgaris* DNA in equine faeces by real-time quantitative PCR. Int. J. Parasitol..

[bib0115] Nielsen M.K. (2012). Sustainable equine parasite control: perspective and research needed. Vet. Parasitol..

[bib0120] Nielsen M.K., Vidyashankar A.N., Olsen S.N., Monrad J., Thamsborg S.M. (2012). *Strongylus vulgaris* associated with usage of selective therapy on Danish horse farms-is it reemerging?. Vet. Parasitol..

[bib0125] Nielsen M.K., Brananb M.A., Wiedenheftc A.M., Digianantonioc R., Garberb L.P., Kopralb C.A. (2018). Parasite control strategies used by equine owners in the United States: a national survey. Vet. Parasitol..

[bib0130] Papini R.A., De Bernart F.M., Sgorbini M. (2015). A questionnaire survey on intestinal worm control practices in horses in Italy. J. Equine Vet. Sci..

[bib0135] Peregrine A.S., Molento M.B., Kaplan R.M., Nielsen M.K. (2014). Anthelmintic resistance in important parasites of horses: does it really matter?. Vet. Parasitol..

[bib0140] R Core Team (2017). R: a Language and Environment for Statistical Computing. https://www.R-project.org/.

[bib0145] Schneider S., Pfister K., Becher A.M., Scheuerle M.C. (2014). Strongyle infections and parasitic control strategies in German horses - a risk assessment. BMC Vet. Res..

[bib0150] Slocombe J.O., McCraw B.M. (1973). Gastrointestinal nematodes in horses in Ontario. Can. Vet. J..

[bib0155] Slocombe J.O., McCraw B.M. (1981). Controlled tests of ivermectin against migrating *Strongylus vulgaris* in ponies. Am. J. Vet. Res..

[bib0160] SJVFS (2010). 17 24 § Legislations (In Swedish) Swedish Board of Agriculture. http://www.jordbruksverket.se/forfattningar/forfattningssamling.

[bib0165] Swedish National Veterinary Institute (2018). Avmaskning Av Häst. http://www.sva.se/djurhalsa/hast/parasiter-hos-hast/avmaskning-av-hast.

[bib0170] Tolliver S.C., Lyons E.T., Drudge J.H. (1987). Prevalence of internal parasites in horses in critical tests of activity of parasiticides over a 28-year period (1956–1983) in Kentucky. Vet. Parasitol..

[bib0175] Uhlinger C. (1993). Uses of fecal egg count data in equine practice. Comp. Cont. Educ. Vet. Pract..

[bib0180] Van Wyk J.A., Mayhew E. (2013). Morphological identification of parasitic nematode infective larvae of small ruminants and cattle: a practical lab guide. J. Vet. Res..

[bib0185] van Doorn D.C., Ploeger H.W., Eysker M., Geurden T., Wagenaar J.A., Kooyman F.N. (2014). Cylicocyclus species predominate during shortened egg reappearance period in horses after treatment with ivermectin and moxidectin. Vet. Parasitol..

[bib0190] von Samson-Himmelstjerna G., Fritzen B., Demeler J., Schurmann S., Rohn K., Schnieder T., Epe C. (2007). Cases of reduced cyathostomin egg-reappearance period and failure of Parascaris equorum egg count reduction following ivermectin treatment as well as survey on pyrantel efficacy on German horse farms. Vet. Parasitol..

